# Ground-based measurements of column-averaged carbon dioxide molar mixing ratios in a peatland fire-prone area of Central Kalimantan, Indonesia

**DOI:** 10.1038/s41598-018-26477-3

**Published:** 2018-05-31

**Authors:** Windy Iriana, Kenichi Tonokura, Gen Inoue, Masahiro Kawasaki, Osamu Kozan, Kazuki Fujimoto, Masafumi Ohashi, Isamu Morino, Yu Someya, Ryuichi Imasu, Muhammad Arif Rahman, Dodo Gunawan

**Affiliations:** 1 Department of Environment Systems, Graduate School of Frontier Sciences, The University of Tokyo, Kashiwa, 277-8563 Japan; 2 Institute for Space-Earth Environmental Research, Nagoya University, Nagoya, 464-8601 Japan; 3 Center for South East Asian Studies, Kyoto University, Kyoto, 606-8501 Japan; 4 Research Institute for Humanity and Nature, Kyoto, 603-8047 Japan; 5 Department of Information Science and Biomedical Engineering, Kagoshima University, Kagoshima, 890-8580 Japan; 6 National Institute for Environmental Studies, Tsukuba, 305-8506 Japan; 7 Atmosphere and Ocean Research Institute, The University of Tokyo, Kashiwa, 277-8568 Japan; 8 Indonesia Agency for Meteorology Climatology and Geophysics (BMKG), Jakarta, 15138 Indonesia

## Abstract

Tropical peatlands in Indonesia have been disturbed over decades and are a source of carbon dioxide (CO_2_) into the atmosphere by peat respiration and peatland fire. With a portable solar spectrometer, we have performed measurements of column-averaged CO_2_ dry-air molar mixing ratios, XCO_2_, in Palangka Raya, Indonesia, and quantify the emission dynamics of the peatland with use of the data for weather, fire hotspot, ground water table, local airport operation visibility and weather radar images. Total emission of CO_2_ from surface and underground peat fires as well as from peatland ecosystem is evaluated by day-to-day variability of XCO_2_. We found that the peatland fire and the net ecosystem CO_2_ exchange contributed with the same order of magnitude to the CO_2_ emission during the non-El Niño Southern Oscillation year of July 2014-August 2015.

## Introduction

Tropical peatland is one of the ecosystems, which stores a large amount of terrestrial carbon over millennia. Indonesian peatlands store 57 Gt C, about 10% of the global peat carbon pool^1^_._ Since the 1970’s, tropical peatlands have been disturbed by logging, anthropogenic land-use change including deforestation and drainage, leading to release of carbon and reduction of carbon storage^2–5^. Peatland fires and oxidative peat decomposition by peat respiration are two main processes that release carbon from peat soil to the atmosphere^3–10^. Tropical peatland is characterized by waterlogged conditions and low rates of decomposition. Drainage construction lowering the ground water level (GWL) enhances aerobic peat oxidation, resulting in steady releases of CO_2_^6,7,11–13^. The estimated amount of carbon emitted from peatlands in Indonesia is 0.1–0.5 Gt C/y, using measured annual flux data of carbon emission. In recent decades, frequency of forest fires across the tropics has been increasing^14^. Forest fires in Indonesia tend to occur annually in the dry season even in non-El Niño Southern Oscillation years primarily by anthropogenic activities for the development of agriculture and plantations^15–18^.

As for assessment of the current status of Indonesian peatland and quantification of its disturbance, the CO_2_ emissions from fires is quantitatively estimated from satellite imagery data by a bottom-up method^3,9,10,19^. This method is robust but has a difficulty in estimating the contribution of smoldering low-intensity underground peatland fires. An alternative top-down method monitors the increments of atmospheric CO_2_ concentrations by satellite observation, *e*.*g*., Greenhouse gases Observing SATellite (GOSAT)^20^.

**Figure 1:**
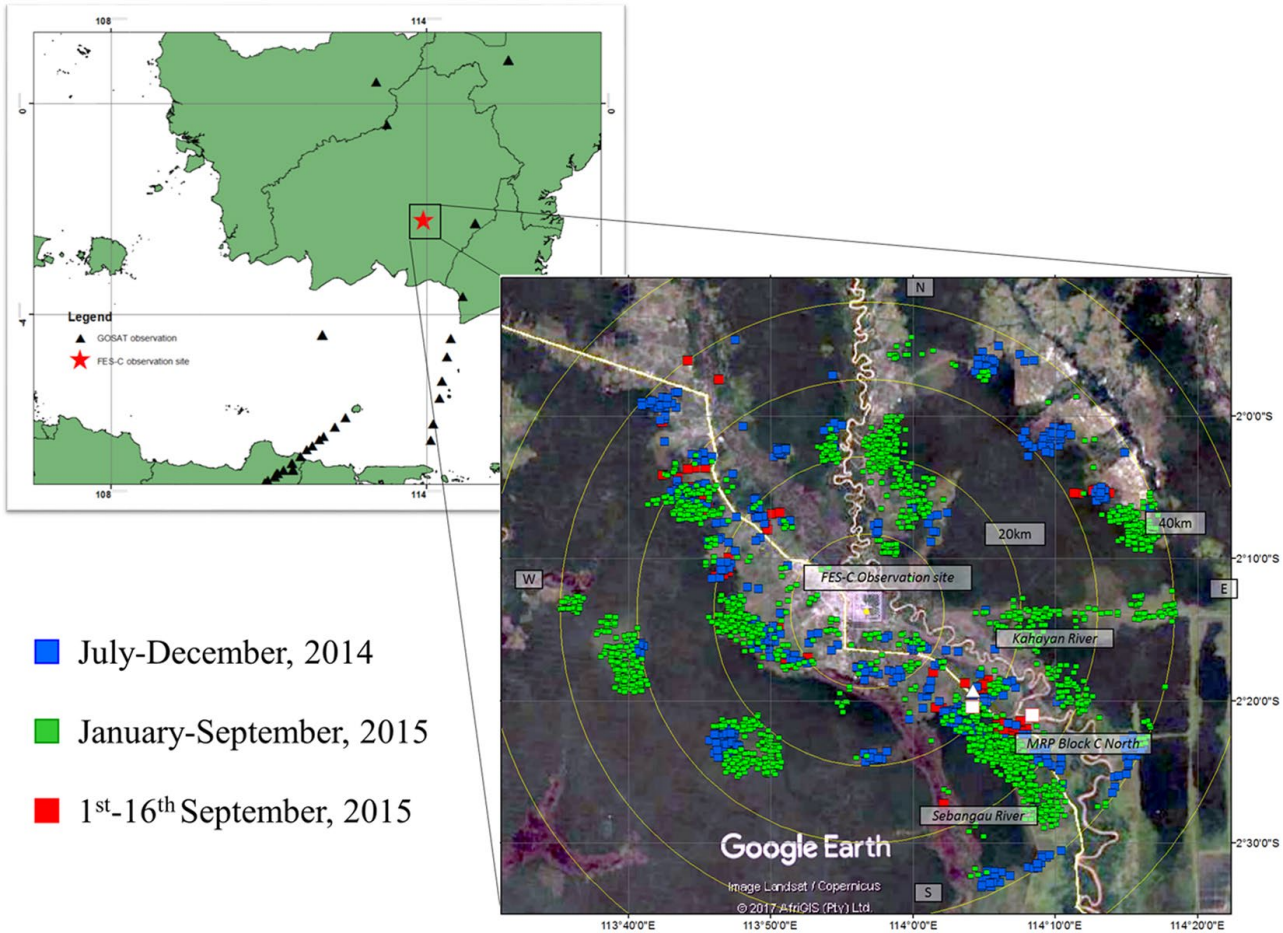
Maps of study field. Right:Hotspot distribution is indicated by colored squares during the fire season. The outer white cicle shows a radius of 50 km. Center location is the Tjilik Riwut Meteorology Station, Palangka Raya, Central Kalimantan, Indonesia, which is the site location for Fiber-Etalon Sun-photometer for column CO_2_, weather radar, airport visibility and AERONET observation. (white square) Eddy covariance site by Hirano *et al*. (ref.^7^). (white triangle) Ground water levlel site by Takahashi *et al*. (ref.^24^). Left: (solid triangle) GOSAT satellite observation points between July 2014-September 2015. (Google Earth, 2017) (ArcGIS 10.2.2 Desktop.10.2.2.3552. 2014. Redlands, CA: Environmental Systems Research Institute).

In this article, we report monitoring of column-averaged dry-air molar mixing ratios, *X*CO_2,_ in an Indonesian fire-prone area over the dry and wet seasons in Palangka Raya, Central Kalimantan, using a portable solar photometer for field measurements. The location of our observation station is shown in Fig. 1. We used a Fiber-Etalon Sun-photometer for column Carbon dioxide (FES-C), which is attached with a fiber Fabry-Perot optical device^21^. It probes larger sample volumes than *in situ* and smaller scales than sensors onboard satellites, and is suitable for measurement of CO_2_ emission from local peatlands.

We discuss variabilities of *X*CO_2_ with use of weather data and peatland-specific parameters including airport visibility records, hotspot counts, GWL data and weather radar images. The MODerate resolution Imaging Spectroradiometer (MODIS) hotspot data^22^ are used to estimate the contribution of surface and underground fires to *X*CO_2_, from which CO_2_ and particulate matter are produced^23^. Peatland fires characterized by low intensity burning spread slowly into peat deposits below the surface for long periods of time, contributing to emissions.

## Results

### Background levels of *X*CO_2_

**Figure 2:**
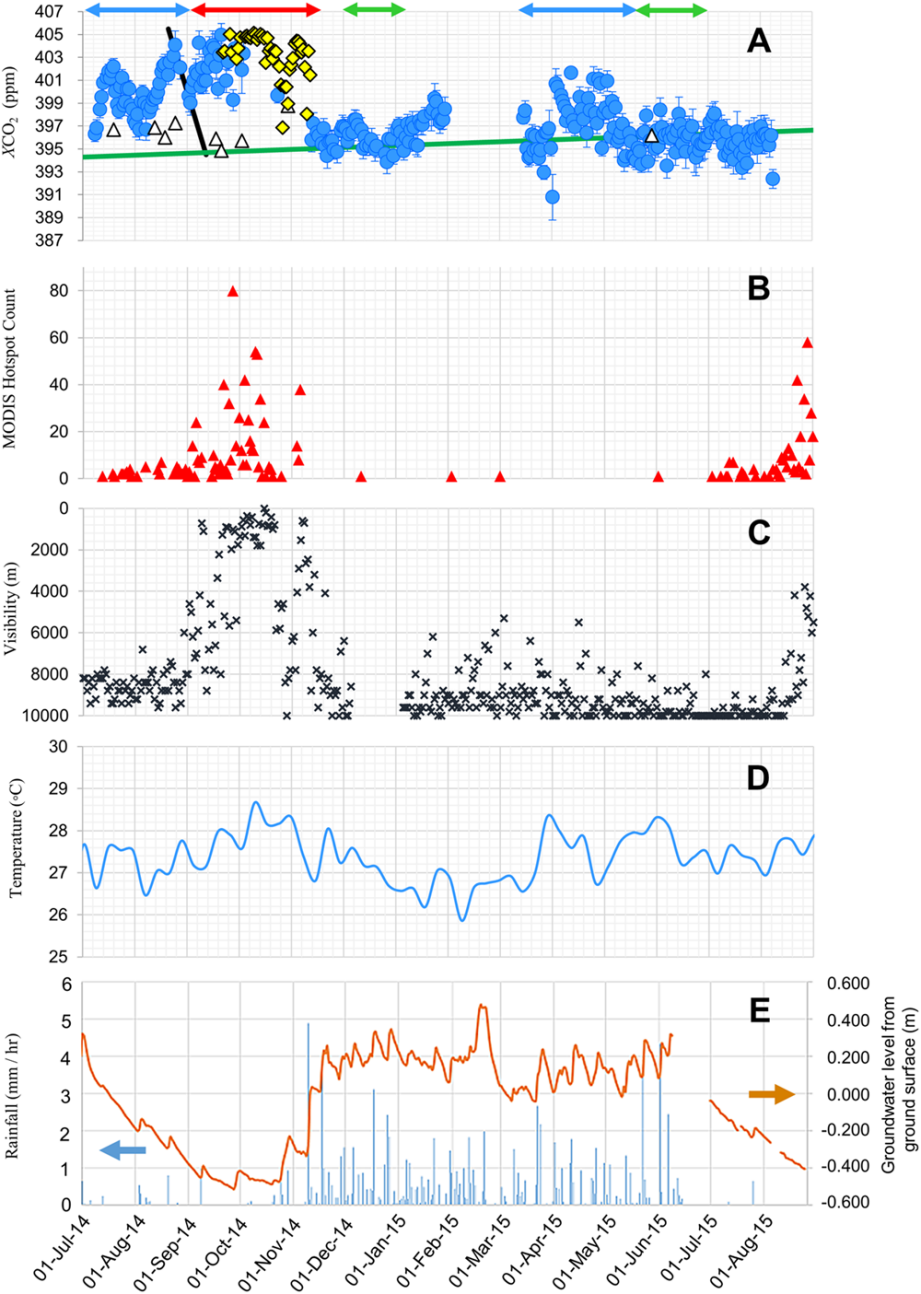
(**A**) Time series of *X*CO_2_; (blue circle) FES-C average for UTC = 3–7 h. The error bar shows the one-standard deviation of *X*CO_2_ value, (∆) GOSAT data on sea, (green line) background level Eq. (1), (yellow ◊) Δ*X*CO_2_ estimated from visibility data by using Eq. (3), (blue ↔ ) peat soil respiration period, (red ↔ ) hot fire period, (green ↔ ) background period. The black solid line indicates the contribution of the peat soil respiration for eye clarity purpose. (**B**) MODIS hotspot count per day in a 50 km circle centered at the BMKG station, (**C**) Airport visibility data presented inversely, (**D**) Weekly average of temperature, (**E**) Daily averages of (blue) precipitation and (brown) ground water level from Takahashi *et al*. (ref.^24^).

A time series of *X*CO_2_ data at Palangka Raya is shown in Fig. 2A along with environmental parameters. Increments in *X*CO_2_ above the background levels, Δ*X*CO_2_, are due to peatland fires and the net ecosystem CO_2_ exchange. Data missing in *X*CO_2_ from mid-September to October 2014 was due to thick haze and dense smoke coverage that weakened the solar spectral intensity.

In estimation of the *X*CO_2_ background levels, we use the annual trend obtained by fitting the GOSAT data on sea between the Kalimantan and Java Islands between 21^st^ July, 2009–30^th^ May, 2015 as described Supplementary S1. The GOSAT annual trend is 2.00 ± 0.10 (1σ) ppm/y. Hereafter, the uncertainty represents one standard deviation. The mean background level of FES-C is 395.8 ppm between 1^st^ December, 2014–6^th^ January, 2015 and 19^th^ May–2^nd^ July, 2015 in non-fire wet seasons that are characterized by high GWL, low temperature and high visibility. At Bukit Kototabang, Sumatra, Indonesia, a meteorological station (0.165°S, 100.295°E) has been performing surface measurement of CO_2_ concentrations that irregularly varied with ±5 ppm due to peat fire and respiration, the mean value of which is 393.4 ppm for the non-fire season between July – November 2014. Combination of the FES-C non-fire wet season data with the annual trend coefficient of 2.00 ppm/y gives the best-fit background level in units of ppm:

1 $$Background\,(n)=(394.3\pm 1.2)+(2.00\pm 0.10)\times \frac{n}{365.25},$$

where *n* is day after 1^st^ July, 2014.

### Increments in *X*CO_2_ by peat respiration

The CO_2_ emission from peat respiration or aerobic peat oxidation has a strong correlation with lowering GWL^7^. As marked by the two blue double arrows in Fig. 2A between July-August 2014 and mid-March –mid-May 2015, Δ*X*CO_2_ increase while mean GWL and precipitation are reduced to −0.12 m and 0.22 mm/h, respectively. The mean temperature goes up by 0.2 °C. During these observation periods, visibility in Fig. 2C is high and no hotspots appear in Fig. 2B. GWL and rain precipitation at the other station, Taruna Jaya (2.23°S, 114.07°E), behaved similarly to those of Fig. 2E^24^. The increments in Δ*X*CO_2_ are due to peat respiration enhancement in peatland and not to fire enhancement.

### Increments in *X*CO_2_ by peatland fires

Figure 2 shows that the fire season in the beginning-to-middle of September 2014 is characterized by high Δ*X*CO_2,_ large hotspot counts, low visibility, low GWL and low precipitation. Δ*X*CO_2_ increased up to 10.0 ppm (mean 6.0 ppm). The increments are caused mostly by peatland fires because the surface peat that is fresh and less decomposed is destroyed by fire and the peat respiration activities are reduced^7^.

## Discussion

### Increments in *X*CO_2_ corrected for airmass transport

Our tethered balloon observation for vertical distribution of the CO_2_ concentrations as described in Supplementary S2 suggests that CO_2_ emitted from the ground into the atmosphere is homogeneously mixed at altitude up to ~700 m and is dispersed by prevailing south-easterly to southerly wind with an average wind reach distances of 160 km/day. (See Supplementary S3) Palangka Raya is located 136 km away from the southerly coastal area and surrounded by lowland peat swamp forest ecosystems^25^. Wind flow that travels over those surroundings reaches daily over Palangka Raya. Using the land-use map and the results of 24-hr backward trajectory calculations, we obtain fractional coverages, *P*_daily_, of the trajectories at 1500 m height travelling over the peatland and lowland from sea area as shown in Supplementary S3. If we assume that the whole peatland area in the southern vicinity of Palangka Raya is homogeneously contaminated by emitted CO_2_, then, the daily emission from peatland may be obtained by Δ*X*CO_2_/*P*_daily_ where *P*_daily_ is the ratio of trajectory distance over peatland /whole trajectory distance. Then, the total molar flux of emission per unit peatland area, *F*, may be obtained from the sum of daily Δ*X*CO_2_/*P*_daily_ during the peat respiration periods of total 122 days in 2014–2015,

2 $$F=Sum({\rm{\Delta }}XC{O}_{2}/{P}_{daily}){{\rm{\sigma }}}_{air},$$

where *Sum* = 715 ± 33 ppm and the molar column density of air, σ_*air*_ = 3.5 × 10^5^ mol/m^2^. The total uncertainty is due to the instrumental and background uncertainties in 122 days as well as *P*_daily_ uncertainty. The uncertainty in *P*_daily_ is estimated to be 25% as described in Supplementary S3.

### Total carbon emission by net ecosystem exchange

The total carbon emission is *F* × *m*_C_ = 3,000 ± 140 gC/m^2^ for two non-fire periods of Fig. 2A in July 2014- May 2015, where *m*_C_ is the molecular weight of carbon (12 g/mol). The yearly net ecosystem CO_2_ exchange controlled chiefly by oxidative peat decomposition, *NEE*, is 1500 ± 70 gC/(m^2^y) averaged for two non-fire seasons in 2014–2015. *NEE* is the difference between the ecosystem respiration and the gross primary production due to photosynthesis. Since this NEE excludes the contributions of emission from lowland and other sources during wind travelling, it should be an upper limit value.

When the contribution of CO_2_ emission from the lowland area is included, *P*_daily_ is defined as the ratio of peatland + lowland trajectory distance/whole trajectory distance: *Sum* = 475 ± 29 ppm and *F* × *m*_C_ = 1,990 ± 120 gC/m^2^, then, *NEE* is 1,000 ± 60 gC/(m^2^y) averaged for 2014–2015. Hirano *et al*.^7^ measured *NEE* per year using the eddy covariance technique on the two locations at both a drained swamp forest (2.35°S, 114.14°E) and a drained burnt swamp forest (2.34°S, 114.04°E) in the former Mega Rice Project area of Palangka Raya. Two stations are located within our observation area. Their *NEE* values show a clear seasonal variation as a function of GWL and range from 105 to 532 (1σ distribution) and from 427 to 571 g C/(m^2^y) over the year of 2004–2008, respectively.

There is a large discrepancy between our estimated *NEE* and Hirano’s reported values. This is partly due to difference in hydrological environments. The average temperature during our observation period in Fig. 2D is higher by ~1.5 °C than during Hirano’s period^7^. According to Luo and Zhou^26^, there are some factors controlling the soil respiration, *e*.*g*., temperature, soil moisture and soil oxygen. In wetlands, oxygen is usually the limiting substrate for respiration, soil drying due to increased temperature could stimulate soil respiration. The discrepancy would also be due to the fact that our measurement is only for one particular observation area during a particular term.

### Correlation between fire-origin *X*CO_2_ and airport operation visibility records to evaluate missing data due to thick haze that weaken the solar spectral intensity

**Figure 3:**
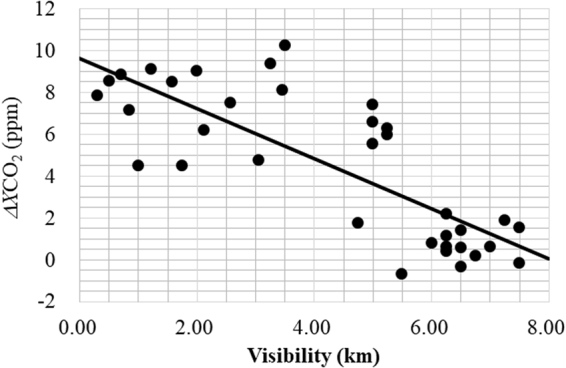
Correlation diagram between Δ*X*CO_2_ and visibility during the fire season of September−November 2014 for Eq. (3). The correlation coefficient is −0.79. Note that visibility was measured in the midnight while *X*CO_2_ in the noon as described in the text. (Power Point 2016).

During the dry season, the area is covered by haze when peatland fires occur, reducing airport visibility. Kusumaningtyas *et al*.^27^ attributed high values of scattering albedo or low visibility to surface and smoldering combustion in the peatland. Using the fire-origin Δ*X*CO_2_ for 35 days of FES-C measurement, a linear relationship between Δ*X*CO_2_ and visibility is empirically obtained with a correlation coefficient *R* = − 0.79 in Fig. 3:

3 $${\rm{\Delta }}XC{O}_{2}=(9.6\pm 0.8)-(1.20\pm 0.16)\times {L}_{vis}$$

where *L*_vis_ is the average visibility in units of km at UTC = 20−0 h as described in Supplementary S4. Several *X*CO_2_ data in September−November are not available because of weak solar intensity due to thick haze conditions, which can be estimated by Eq. (3). Thus obtained *X*CO_2_ are plotted by the yellow diamonds in Fig. 2A. Using both the measured and calculated values, we estimated the mean <Δ*X*CO_2_> to be 7.8 ppm during the fire season from 3^rd^ September, 2014 to 11^th^ November, 2014, total 70 days.

### Haze area size of air contaminated with emitted species

**Figure 4:**
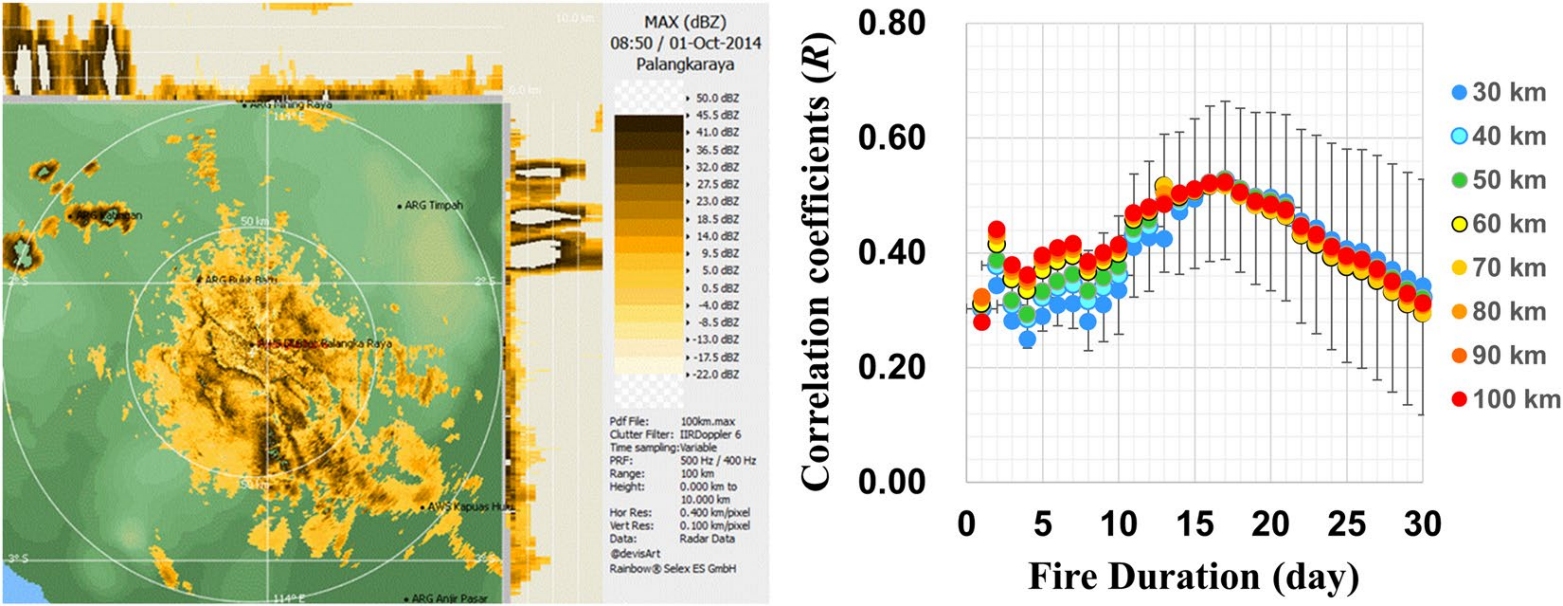
Left: Typical haze area image of C-band radar at UTC 8:50 on 6^th^ October, 2014. The white outer circle shows a radius of 100 km. The white scale on the side is 2.5 km height /div. Right: Correlation coefficients (*R*) between Δ*X*CO_2_/*P*_daily_ and the distance-normalized hotspot count (1/*r*^2^) accumulated for active duration of underground fire. *r* is the distance between a hotspot and the observation station. In the abscissa, the number includes one day of surface fire. Distances are color-coded every 10 km for the 30–100 km range. Light blue circles for 40 km are shown with one-standard deviation. The analysis period is between 3^rd^ September−11^th^ November, 2014. (Power Point 2016).

We use the data of Fig. 2 in evaluating peatland dynamics caused by fire in combination with the data from satellite observation. Comparing Fig. 2B,C, one notices that visibility is low about two more weeks after surface fire ceases. Aerosol optical depth (AOD) images from the Terra-MODIS AOD data base^28^ show dense aerosol plumes over Palangka Raya with a size of 30–100 km, an example of which is shown in Supplementary S5. To estimate quantitatively the sizes of hazy areas over Palangka Raya, we inspect our radar images measured with a C-band Doppler weather radar at the station during the fire period between 15^th^ September – 11^th^ October, 2014. During the period, precipitation is below 10 mm/month and the AODs reported by the AERONET are over three^29^, suggesting that the radar images are due to fire-origin aerosols. A typical haze area image over Palangka Raya is shown in Fig. 4(left). The average radius of 48 images is 44 ± 14 km with height below 1.6 km. We assume that the air is homogeneously mixed and flows from the fire clusters all around the vicinity of Palangka Raya in Fig. 1. According to the NASA multi-angle imaging spectroradiometer analysis of fire plumes in the Borneo Island by Zender *et al*., fire plumes travel with the mean length of 41.4 ± 26.7 km and the average plume width-to-length ratios of 0.268 ± 0.147^30^.

### Estimation of active duration of underground fires from correlation between hotspot count and increment in *X*CO_2_

Burning of peat soil occurs under smoldering condition characterized by low heat and long duration that can last up to four months^3,9,31,32^. Hence, it is essential to estimate an active duration of the underground fire. For quantitative analysis, the hotspot data between September−November 2014 are chosen. Supplementary S6 shows that the hotspot count density over peatland is evenly distributed where hotspot counts 506−4186 are selected to analyze with high confidence. Since prevailing southerly wind travels with an average reach distance of 160 km/day during the fire season, daily Δ*X*CO_2_ is corrected by the daily trajectory fraction *P*_daily_ over peatland. Below we evaluate correlation coefficients, *R*, between Δ*X*CO_2_/*P*_daily_ and hotspot count accumulated for active durations of underground fires as described in Supplementary S6. In this data analysis, the contribution of a hotspot to Δ*X*CO_2_/*P*_daily_ is distance-normalized by 1/*r*^2^ where *r* is the distance between the hotspot and the observation station. To simplify the analysis, we assume the following conditions: 1) surface fire with high temperature is detected by the MODIS satellite sensor and lasts for one day as a hotspot, 2) underground fire lasts longer but undetectable by the MODIS sensor as described in Method, and 3) hotspots emit the same amount of CO_2_ until emission ceases.

The results of correlation analysis are shown in Fig. 4(right), in which *R* depend on the active underground fire duration of 1–30 days (including one day of surface fire) but does not on the observing fire field ranges of *r* = 30–100 km radii. The correlation coefficients are independent of *r* because hotspots distribute evenly over the peatland area. We found *R* ~ 0.5 with an active fire duration centered ~17 days (including one day of surface fire) for all *r*. Within the range of one-standard deviations shown in Fig. 4, the active fire duration is estimated to be in a range of 10–25 day centered around 17 days.

The fire image sensor on board the TET-1 satellite has detection capabilities in monitoring low-intensity peatland fire fronts through smoke. Fire front edges observed in the TET-1 images may be indicative that the fire is slowly spreading through the deeper peat layer for two weeks in the peatland of Central Kalimantan^33^.

### Total emission of CO_2_ per MODIS hotspot

During the fire season, *Sum*(Δ*X*CO_2_/*P*_daily_) is 886 ± 33 ppm. In this calculation, we include Δ*X*CO_2_ values estimated from Eq. (3) for the thick haze period. The uncertainty includes uncertainties of instrumental and estimation of ΔXCO_2_ in Eq. (3) as well as that of *P*_daily_. We obtain the conversion factor *CF* for emission per hotspot by;

$$CF=Sum({\rm{\Delta }}XC{O}_{2}/{P}_{daily}){{\rm{\sigma }}}_{air}S(r)/N(r).$$

where *S*(*r*) = π*r*^2^ is the observing fire area, and *N*(*r*) is the total hotspot count during the fire season. According to the haze images, we estimate the active observation area for XCO_2_ to be *r* = 44 ± 14 km. In the range of 30–60 km, the hotspot density, *N*(*r*)/*S*(*r*), is almost constant 0.16 ± 0.02 count/km^2^, due to homogeneous distribution of the hotspots over the peatland. Thus calculated conversion factor during the fire season of 2014 in Palangka Raya is *CF* = (8.5 ± 1.4) × 10^7^ kgCO_2_/hotspot.

## Summary and Conclusion

We have demonstrated the approach of using solar absorption spectra recorded with a small Fabry-Perot solar photometer at a site in a source region for an estimation of the CO_2_ source strength of the surrounding fires. This methodology is applied to observation of the variability of the column averaged dry-air molar mixing ratios of CO_2_ in Palangka Raya, Central Kalimantan, Indonesia in July 2014−August 2015, an non-ENSO year. Our methodology is not a general one but a specific one to peatland field measurements. Assuming the conditions described in the text, the CO_2_ data are analyzed in combination with the peatland-specific parameters such as hotspot counts, water levels, airport visibility records and radar images as well as weather data. We found that the increments are due to the underground smouldering fires in the fire season and the aerobic peat oxidation in the beginning of the dry season. Both sources contribute with the same order of magnitude to the enhancement in the atmospheric CO_2_ concentration.

## Methods

### Location of the observation station and its surroundings

Monitoring of *X*CO_2_ was performed at the Tjilik Riwut Palangka Raya BMKG Station, Palangka Raya, Central Kalimantan, Indonesia (2.224°S, 113.946°E, 10 m a.s.l.) from June 2014 to August 2015 with a portable Fiber-Etalon Sun-photometer for CO_2_, instrumental details of which are described in Supplementary S7. Visibility records and weather data were provided by the BMKG station. The station for measurement of precipitation and ground water level is located at 2.31°S and 114.06°E as shown in Fig. 1^24^. The C-band Doppler weather radar (Gematronik/Selex SI, Meteor 600 C, single / linear horizontal polarization) was located at the Palangka Raya BMKG station.

### Satellite product 1: hotspot counts

We use daily active fire detection data derived from the MODIS observation as a peatland fire index, extracting the data collection 6 from the Fire Information for Resources Management System^22^. The MODIS active fire product includes the hotspot counts with high-confidence detection in a range larger than 60%. In MODIS active fire algorithm, temperature thresholds of detection potential fire pixel is 300–314 K^34^. Although the temperature of underground fire reaches above them, the spatial resolution and thick haze hamper the ability of the MODIS sensor to detect some of active smouldering fires.

### Satellite product 2: carbon dioxide mixing ratios of GOSAT

The GOSAT satellite data set is a product of measurements on the Java Sea between the Kalimantan and Java Islands as shown in Fig. 1(left). GOSAT data (V02.21) from 15^th^ June, 2009 to 23^rd^ April, 2014, (V02.31) from 25^th^ June, 2014 to 29^th^ October, 2014, (V02.40) from 6^th^ May, 2015 to 29^th^ July, 2015.

### Data Availability

Datasets during the current study are available in http://www.tonokura-lab.k.u-tokyo.ac.jp/pub/SR.html.

## Electronic supplementary material

supplementary information
