# Supplementary material for: Ground-based measurements of column-averaged carbon dioxide molar mixing ratios in a peatland fire-prone area of Central Kalimantan, Indonesia
Source: Sci Rep. 2018 May 31;8:8437. doi: 10.1038/s41598-018-26477-3 (PMC5981433; doi:10.1038/s41598-018-26477-3)
Supplement: Supplementary file 1 — supplementary information [file 41598_2018_26477_MOESM1_ESM.docx]

**Supplementary Information**

**Ground-based measurements of column-averaged carbon dioxide molar mixing ratios in a peatland fire-prone area of Central Kalimantan, Indonesia**

**Windy Iriana^1^, Kenichi Tonokura^1^, Gen Inoue^2^, Masahiro Kawasaki^2,3,4,†^, Osamu Kozan^3,4^, Kazuki Fujimoto^5^, Masafumi Ohashi^5^, Isamu Morino^6^,** **Yu Someya^7^, Ryuichi Imasu^7^, Muhammad Arif Rahman^8^ & Dodo Gunawan^8^**

^1^Department of Environment Systems, Graduate School of Frontier Sciences, The University of Tokyo, Kashiwa 277-8563, Japan. ^2^Institute for Space-Earth Environmental Research, Nagoya University, Nagoya 464-8601, Japan. ^3^Center for South East Asian Studies, Kyoto University, Kyoto 606-8501, Japan. ^4^Research Institute for Humanity and Nature, Kyoto 603-8047, Japan, ^5^Department of Information Science and Biomedical Engineering, Kagoshima University, Kagoshima 890-8580, Japan. ^6^National Institute for Environmental Studies, Tsukuba 305-8506, Japan. ^7^Atmosphere and Ocean Research Institute, The University of Tokyo, Kashiwa 277-8568, Japan. ^8^Indonesia Agency for Meteorology Climatology and Geophysics (BMKG), Jakarta 15138, Indonesia. Correspondence and requests for materials should be addressed to M.K. (email: kawasaki@moleng.kyoto-u.ac.jp)

**CONTENTS**

**Item Title**

|  | Text S1  Figure S1 | Estimation of XCO_2_ background levels from GOSAT data  Fitting results to GOSAT *X*CO_2_ data |
| --- | --- | --- |
|  | Text S2 | Tethered balloon measurements of *in situ* CO_2_ concentration and weather data |
|  | Figure S2 | *In-situ* balloon measurements; CO_2_ concentration, wind speed and wind direction |
|  | Text S3  Figure S3-1  Figure S3-2  Figure S3-3  Figure S3-4 | Wind speed, land-use map and backward trajectories of wind flow in Central Kalimantan  Temporal distributions of prevailing southerly wind speed  Land-use map and 24-hr backward trajectory of wind flow in Central Kalimantan  Daily emission of CO_2_ during fire season  Daily emission of CO_2_ during non-fire season |
|  | Text S4 | Airport operation visibility records used for analysis |
|  | Figure S4-1 | Daily average *<X*CO_2_> and airport visibility data |
|  | Figure S4-2 | Correlation coefficients between *X*CO_2_ and visibility as a function of temporal offsets between the two observation times/ Running averaged temperature, humidity and wind speed |
|  | Figure S4-3 | Aerosol optical depth at 500 nm *vs.* visibility |
|  | Text S5  Figure S5 | Haze area images from Terra-MODIS observation and C-band Doppler radar measurements  Terra-MODIS AOD and radar images |
|  | Text S6  Table S6  Figure S6 | Hotspot distribution around the observation site and correlation analyses  MODIS hotspot counts and hotspot density in 2014  Example of the correlation diagrams |
|  | Text S7 | Instrumentation |
|  | Figure S7-1  Figure S7-2 | Schematic diagram of data acquisition system  Transmission spectrum of the fiber Fabry-Perrot interferometer optics |
|  | Figure S7-3 | Example of calibration curves for conversion of observed optical density to *X*CO_2_ |
|  | Figure S7-4  Figure S7-5 | Daily averaged data of FES-C and FTS at Tsukuba, Japan  Column averaging Kernel |

**Text S1*Estimation of XCO_2_ background levels from GOSAT data***

Figure S1 shows GOSAT *X*CO_2_ data above the Java Sea, from 21^st^ July, 2009 to 30^th^ June, 2015 ^1^. GOSAT data scatter above and below the best-fitted trend line, *Tr*(*n*) in ppm, with a coefficient of 2.00±0.10 ppm/y (1σ) and intercept STDs of 10.7 ppm (1σ),

$Tr\left( n \right)=385.2\pm10.7+\left( 2.00\pm0.10 \right)\times\frac{n}{365.25}$ (S1)

where *n* is day after January 1^st^, 2009. This large intercept STD is due to natural variation in XCO_2_ as described in the main text.

A best-fitted seasonal cycle formula is shown by the solid curve^2^.

, (S2)

where *t* (month) starts from January 1^st^, 2009.

| Intercept | 385.8 ppm | Amp_1_ | 2.8 ppm | Amp_2_ | 1.4 ppm |
| --- | --- | --- | --- | --- | --- |
| Trend | 2.00 ppm/y | *φ*_1_ | 4.7 month | *φ*_2_ | 10.6 month |


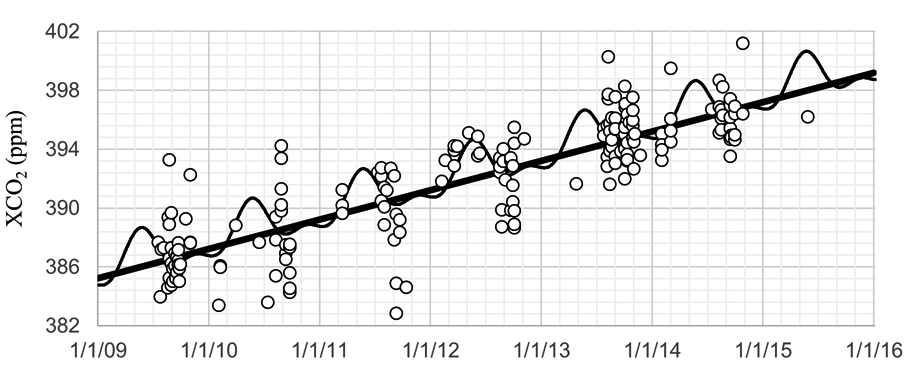


Fig. S1. Fitting results to GOSAT *X*CO_2_ data**. Circle**: GOSAT *X*CO_2_ data above the Java Sea. **Solid line**: trend line of Eq. S1, **Solid curve**: seasonal cycle of Eq. S2. (Power Point 2016)

As seen in Fig. S1 and also described in the main text, the GOSAT XCO_2_ data scatter so much. To reduce the error range of the intercept of the best-fit trend line, we combine the FES-C non-fire wet season data with the GOSAT annual trend coefficient of 2.00 ppm/y gives the best-fit background level in units of ppm:

$Background\left( n \right)=(394.3\pm1.2)+\left( 2.00\pm0.10 \right)\times\frac{n}{365.25}$ , (S3)

where *n* is day after 1^st^ July, 2014.

**Text S1 References**

1. Greenhouse Gases Observing Satellite data, GOSAT data (V02.21) from 15^th^ June, 2009 to 23^rd^ April, 2014, (V02.31) from 25^th^ June, 2014 to 29^th^ October, 2014, (V02.40) from 6^th^ May, 2015 to 29^th^ July, 2015,

GOSAT Data Archive Service (GDAS), https://data2.gosat.nies.go.jp/index_en.html

NIES GOSAT Project, https://data2.gosat.nies.go.jp/doc/documents/ReleaseNote_FTSSWIRL2_V02.x5_en.pdf

2. Miyamoto. Y. *et al*. Atmospheric column-averaged mole fractions of carbon dioxide at 53 aircraft measurement sites. *Atmos. Chem. Phys*., **13**, 5265–5275, doi: 10.5194/acp-13-5265-2013 (2013).

**Text S2 *Tethered balloon measurements of in situ CO_2_ concentrations and weather data***

Measurements were performed with a tethered balloon (1.5 m dia., 6 m long) on 21^st^–22^nd^ August, 2011 at the Palangka Raya peatland. Figure S2 shows that *in situ* CO_2_ concentrations and wind speed were almost uniform up to 700 m altitude. The air was homogeneously mixed vertically by southerly wind. Note that the fluctuations of the data were caused by vibration of the balloon cargo box. The following devices were on board for measurements.

**Item Supplier Model**

Concentration SenseAir K30

of CO_2_

Temperature Syscom SHT71, SHTD, SHTDA-2-L-1M

Wind speed Kanomax 0964-01

Wind direction Akizuki TDS01V

Pressure Setra 760

Data transfer MaxStream X-Stream





Fig. S2. *In-situ* balloon measurements; CO_2_ concentration, wind speed and wind direction. **Upper:** morning, **Lower:** afternoon. Note that CO_2_ concentrations are biased by −30 ppm.

**Text S3 *Wind speed, land-use map and backward trajectories of wind flow in Central Kalimantan***

Wind speed temporal changes at Palangka Raya Airport are shown in Fig. S3-1 for non-fire and fire seasons. The data are 4hr-running averaged around center time. The average wind speed is 1.9 m/s, corresponding to 160 km/day.

Figure S3-2 shows the land-use map in the vicinity of Palangka Raya and the coastal area^1^. 24-hr backward trajectory traces of 1500 m altitude are overlaid, which are taken from NOAA HYSPLIT MODEL/GDAS calculation^2^. Fractional coverages, *P*_daily_, of the trajectories travelling over the peatland are calculated during the fire and non-fire season, the mean values and 1σ distribution ranges of which are 0.67±0.28 and 0.64±0.27, respectively. In the non-fire season, if the lowland area is included, the mean value and 1σ distribution range of *P*_daily_ over peatland+lowland are 0.90±0.19.

To calculate *Sum*( Δ*X*CO_2_/ *P*_daily_) we plot the daily data in Fig. S3-3 and -4, in which we eliminate 1) abnormal data that deviate over 2σ from the averaged value and 2) wind speed exceeds over 6 m/s. 9-days of data are eliminated from total 70-days for fire season, 15-day (*P*_daily_ over peatland) and 13-days (*P*_daily_ over peatland+lowland) from total 122 days for non-fire season. Elimination is due to too long trajectory distance or northerly wind conditions. The data eliminated are replaced by interpolated values. The average value of Δ*X*CO_2_/ *P*_daily_ is 12.7 ppm/day for fire season. The values for non-fire season are 5.9 and 3.9 ppm/day under the trajectory conditions over peatland and peatland+lowland, respectively.

A tracer study for trajectory models reported a root-mean-square trajectory error of ~200 km for the 800 km transit distance on the north America area, that is, the uncertainty is 25%^3^. In the present paper we assume the uncertainty in Δ*X*CO_2_/*P*_daily_ is 25%.





Fig. S3-1 Temporal distributions of prevailing southerly wind speed. The horizontal bold line stands for average speed. **A**: 1^st^ July till 31^st^ August, 2014, **B**: 8^th^ September till 15^th^ November, 2014, **C**: 15^th^ March till 14^th^ May, 2015


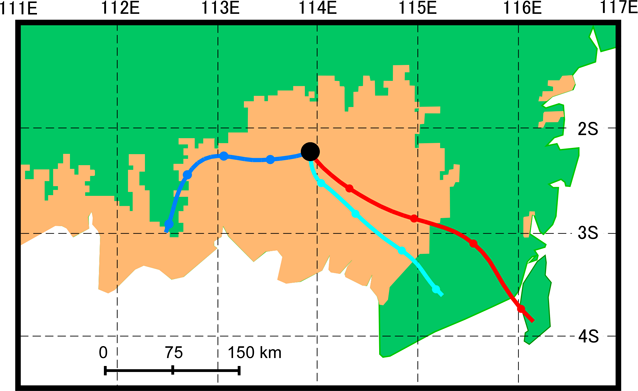


Fig. S3-2. Land-use map, (**Apricot**): peatland, (**Green**):lowland, (**White**): sea^1^. The black circle is the meteorological station. Solid curves: Examples of backward trajectories of 1500 m altitude from NOAA HYSPLIT MODEL calculation^2^. **(Light Blue line)**: 23rd July, 2014 non-fire period and *P*_daily_(peat) = 0.75, *P*_daily_(peat+lowland) = 1.00, (**Red line)**: 30th Sept. 2014 fire period and *P*_daily_(peat) = 0.48, *P*_daily_(peat+lowland) = 0.95. (**Deep** **Blue line)**: 20th Mar, 2015 non-fire period and *P*_daily_(peat) = 0.65, *P*_daily_(peat+lowland) = 1.00. (Canvas ver. 15.5 build 1809)





Fig. S3-3 Fraction normalized daily emission of CO_2_ during fire season. Fractional coverage *P*_daily_ is evaluated from trajectory length over peatland.

^

^

Fig. S3-4 Fraction normalized daily emission of CO_2_ during non-fire season. () fractional coverage *P*_daily_ are evaluated from trajectories over peatland, and (O) over peatland + lowland. Note that some data points with *P*_daily_ =1 are overlapped.

**Text S3 References**

1. Marlier, M. E. et al. Regional air quality impacts of future fire emissions in Sumatra and Kalimantan, *Environm. Res.Lett.* **10**, 054010 (2015): DOI 10.1088/1748-9326/10/5/054010

2. Air Resources Laboratory, NOAA's Office of Atmospheric Research, National Oceanic and Atmospheric Administration, U. S. A. https://ready.arl.noaa.gov/hypub-bin/trajtype.pl (2018)

3. Haagenson, P. L., Kuo, Y-H. & Skumanich, M. *J. Clim. Appl. Meteor.* 26, 410,

doi:10.1175/1520-0450(1987)026<0410:TVOTM>2.0.CO;2 (1987)

**Text S4 *Airport operation visibility records***

Figure S4-1 shows daily average <*X*CO_2_> and airport visibility records. Visibility, *L*_vis_, is a measure of the transparency of the atmosphere, and is defined as the greatest distance at which a target object can be recognized against the horizon sky night and day^1^. Field et al. used visibility records from Kalimantan’s Indonesia Agency for Meteorology Climatology and Geophysics (BMKG) stations at airports, finding an excellent correspondence between the extinction coefficients and total particulate matter emission from fires ^2^.

In the following, we use visibility data from the Tjilik Riwut BMKG Station between 20^th^ August−29^th^ November, 2014. Peatland fires both on the surface and underground emit CO_2_ as well as particulate matter night and day. Figure S4-2 shows a correlation between daily <*X*CO_2_> running averaged for UTC = 3–7 h and airport visibility data with temporal offsets of observation time for visibility, in which the airport visibility data are running averaged for four hrs around each observation time. We assume that the extension coefficient is constant around a fixed observation point. The strongest correlation is obtained when visibility data at UTC = 22 h one day ahead of the <*X*CO_2_> observation time. Before sunrise at UTC ~ 23 h under the weather conditions of slow wind, low temperature and high humidity, aerosol sizes of a shallower mixing layer become larger to reduce visibility due to light scattering since the long-lasting underground fire continues emission of both fine particles and CO_2_. Note that the time lag between UTC and local time is 7 h.


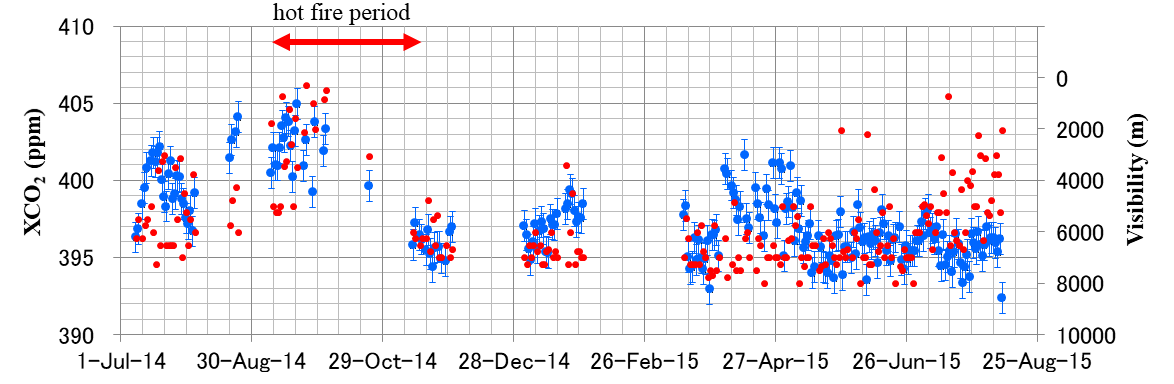


Fig. S4-1. Daily average <*X*CO_2_> and airport operation visibility data. ●; <*X*CO_2_> averaged for UTC = 3-7 h in units of ppm (left-side ordinate), ●; visibility in units of m (right-side ordinate) averaged for UTC = 20-0 h one-day ahead. The vertical bars represent daily variation (one sigma) (Power Point 2016)





Fig. S4-2. **Upper**: Correlation coefficient, *R,* between <*X*CO_2_> and four-hr running averaged airport visibility data with temporal offsets between the observation time for visibility and *X*CO_2_ during the fire period marked by the horizontal double arrow in Fig. S4-1. **Middle**: four-hr running averaged airport temperature and humidity data. **Lower**: four-hr running averaged airport wind speed. (Power Point 2016)

It is interesting to note that visibility and aerosol optical density (AOD) have a strong correlation as shown in Fig. S4-3. The data of AOD at 500 nm are obtained from the AERONET site ^3^.





Fig. S4-3. Aerosol optical density at 500 nm (Ref. 3) *vs.* average visibility measured at UTC = 3–7 h under simultaneous measurements at Palangka Raya from 19^th^ August, 2014 till 30^th^ November, 2014. The correlation coefficient, *R*, is −0.91. (Power Point 2016)

**Text S4 References**

1. Martin, L. C., Boys, R. V., van Donkelaar, B. L., Ruzzante, A., S. Evaluation and application of multi-decadal visibility data for trend analysis of atmospheric haze, *Atmos. Chem. Phys*., **16**, 2435–2457, doi: 10.5194/acp-16-2435-2016 (2016).
2. Field, R. D., van der Werf, G. R. & Shen, S. P., Human amplification of drought-induced biomass burning in Indonesia since 1960, *Nat. Geosci.*, **2,** 185–188, doi:10.1038/ngeo443 (2009).
3. Holben B., NASA, U. S.A., AERONET Version 2 Direct Sun Algorithm, https://aeronet.gsfc.nasa.gov/cgi-bin/type_one_station_opera_v2_new?site=Palangkaraya&nachal=2&level=3&place_code=10 (2017)

**Text S5 Haze area *images from Terra-MODIS observation and C-band Doppler radar measurements***

An AOD image in Fig. S5(left) shows that a dense haze cloud covers the area of Palangka Raya with a radius of 30-70 km and is shifted toward the northern area due to the southern / southeastern wind ^1^. The dense haze area indicated by the brawn color is in fair agreement with the estimated range of the hazy areas below.

We have measured aerosol images with a C-band Doppler radar (Gematronik/Selex SI, single/linear horizontal polarization) at Palangka Raya BMKG station during the fire period for 15^th^ September– 11^th^ October, 2014 when precipitation is below 10 mm/month and the AODs of AERONET are over three. A typical image is shown in Fig. S5 (**Right**). The average hazy area radius of the fifty radar aerosol images is 44 km with a 1σ range of 14 km. The maximum hazy area heights in most images are below 1000 m.


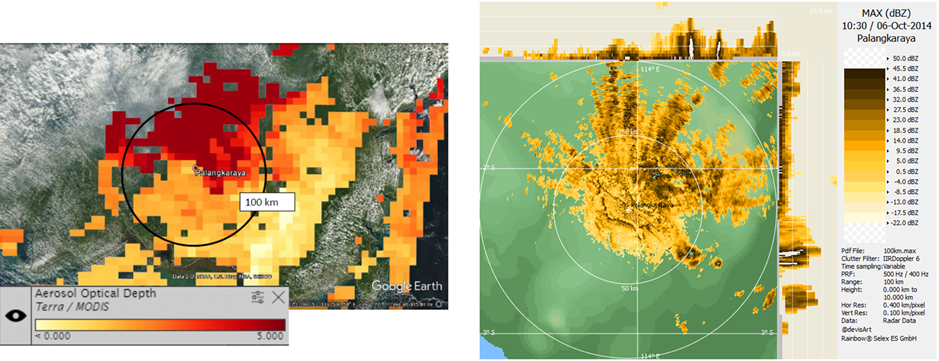


Fig. S5. **Left**: Terra-MODIS AOD image on 20^th^ Sept., 2014 from Ref.1. The black circle has a radius of 100 km. **Right**: haze area image with a C-band Doppler radar of the BMKG station on 6^th^ October, 2014. The white outer circle corresponds to a radius of 100 km. Each white line on the side corresponds to 2.5 km height.

**Text S5 Reference**

1. MODIS Aerosol Optical Depth layer uses the Optical Depth Land And Ocean parameter from both the Terra (MOD04_L2) and Aqua (MYD04_L2), NASA, U.S.A. https://worldview.earthdata.nasa.gov/(2017).

**Text S6 *Hotspot distribution around the observation site and correlation analysis***

Total hotspot counts during entire fire season, *N*(*r*_max_), under confidence level > 60 are listed in Table S6 as a function of distance, *r*_max_, from the observation station. The hotspot densities, *N*(*r*_max_)/π*r*_max_^2^, are almost constant for *r*_max_ = 30-100 km, suggesting a homogeneous distribution of the hotspots over the peatland in the vicinity of Palangka Raya.

Correlation coefficients, *R*, in Fig. 4(Right) in the main text are obtained from correlation analyses between Δ*X*CO_2_/*P*_daily_ and the hotspot duration parameter, *HP*:

*HP*(d_f_, d_i_, r_max_) = Σ*_d_* (Σ*_r_ n*(*r*,*d*)/*r*^2^): summation over *r =* 0~ r_max_ and *d* = d_i_~d_f_ (S-6)

where *n*(*r*,*d*) is daily hotspot count at *r.* d_f_ is an observation date for Δ*X*CO_2_/*P*_daily_. The difference, Δ*d* = d_f_ − d_i_, is an active duration of underground fire in units of day, which varies from 0 to 29 days in Fig. 4(Right). Examples of correlation diagrams are shown in Fig. S6 for *r* = 40 km and Δ*d* = 0 (only surface fire) and 16 days (underground fire lasts for 16 days), in which *R* are 0.32 and 0.52, respectively.

Table S6. MODIS hotspot counts from Ref. 1 and hotspot density in 2014

| Distance, *r* (km) | 100 | 90 | 80 | 70 | 60 | 50 | 40 | 30 |  |
| --- | --- | --- | --- | --- | --- | --- | --- | --- | --- |
| Total Hotspot count, *N* | 4186 | 3644 | 3206 | 2619 | 1978 | 1193 | 712 | 506 |  |
| Density, *N*/π*r*^2^ (1/km^2^) | 0.13 | 0.14 | 0.16 | 0.17 | 0.17 | 0.15 | 0.14 | 0.18 |  |


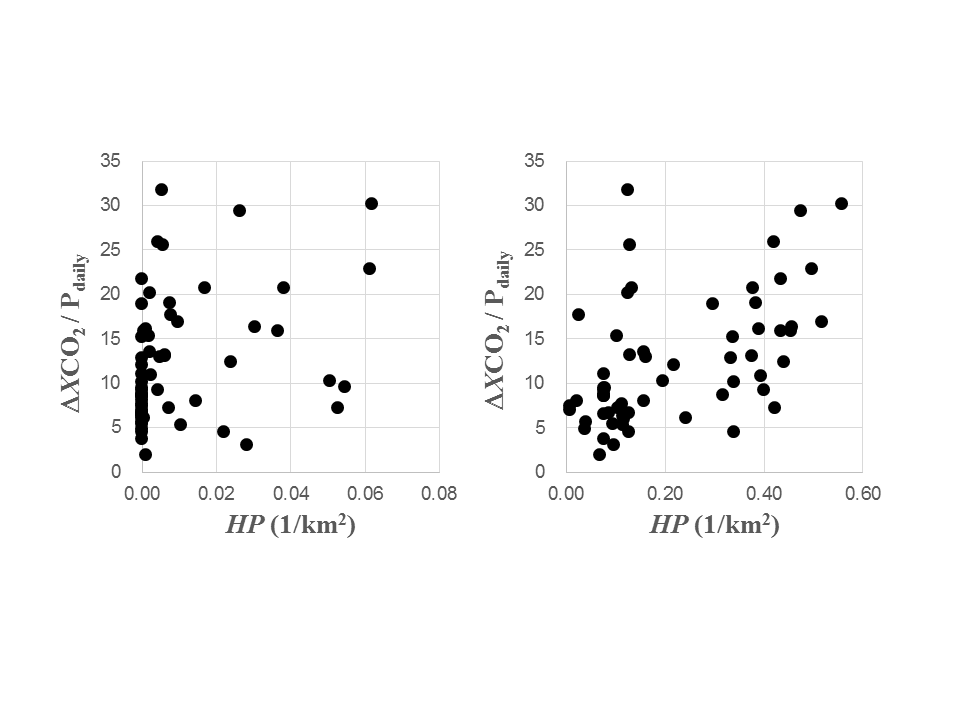


Fig. S6. Example of the correlation diagrams for Eq. S-6. *r* = 40 km and (**Left)** Δ*d* = 0 day and (**Right)** 16 days.

**Text S6 Reference**

1. MODIS active fire product includes the hotspot numbers with high-confidence detection in a range larger than 60%. Masuoka, E., Firms Group, NASA, U.S.A., Fire Information for Resource Management System, https://firms.modaps.eosdis.nasa.gov/download/(2017).

**Text S7 *Instrumentation***

To measure atmospheric CO_2_ column density, a Fabry-Perot interferometer made of quartz glass has a high enough spectral resolution for resolving the rotational photoabsorption lines centered at 1575 nm ^1^. Our portable instrument consists of a ﬁber interferometer developed for use in telecommunication industry. Details are described in our previous paper ^2^. A schematic diagram of the data acquisition system is shown in Fig. S7-1. In brief, outdoor devices consist of a fiber collimator (Thorlab, F280FC-155) as a small solar telescope installed on a portable sun tracker (Meisei Electronics, slant movement with time resolution of 1 sec) and a Global Positioning System (GPS) device (Position, GPS-74A). Indoor devices consist of the ﬁber Fabry-Perot interferometer (FFPI, Nippon Electric Glass, single mode optical fiber of 13 mm long and 1.25 mm diameter), a solar intensity monitor and a laptop computer. A long-pass filter (Thorlab FEL1500, and Edmond NT32-760) is placed in front of an object lens of the solar telescope. The solar signal is guided to the FFPI and the solar intensity monitor via a single-mode optical fiber cable (Nittetsu-Sumitomo, MES-32495-02, 9 μm dia.) attached with a beam separator. As shown in Fig. S7-1, one fiber is connected to the signal inlet of the FFPI while the other to that of the solar intensity monitor. Reference solar intensity is monitored in the spectral range of 800–1800 nm simultaneously with the FFPI signal to compensate the signal for solar intensity fluctuation caused by thin cloud coverage of the solar light. The wavelength of the solar spectrum transmitted through the FFPI is controlled by changing its temperature. The transmitted light is thus aligned/unaligned with the CO_2_ rotational lines that are centered at 1572 nm through a narrow band fiber filter (Fiber Labs, DWDM-1-2-07-900-1-0.3-FA). The transmission spectrum of the FFPI is shown in Fig. S7-2.

By modulating the FFPI temperature 40 s/cycle, the intensity ratio of the incident to transmitted light is deduced by using the Beer-Lambert law, which relates the absorption of light to the properties of the material through which it is traveling. As will be described in the following, we convolute the optical transmission spectrum of the FFPI optics with the spectra simulated with use of weather data for various CO_2_ mixing ratios. This allows CO_2_ columns to be measured by simulating CO_2_ spectra once one knows optical characteristics of the FFPI and weather conditions of the solar light path.

Figure S7-1. Schematic diagram of data acquisition system





Fig. S7-2 Transmission spectrum of the fiber Fabry-Perrot etalon optics. Free Spectral Range = 0.324 nm (1.25 cm^-1^),  Full-Width at Half Maximum = 0.025 nm (0.10 cm^-1^)

***Column averaged dry-air molar mixing ratios*** After the solar light passes through the atmospheric layer and interacts with the gas molecules, the rovibronic bands have lower intensity due to absorption by CO_2_ in the near infrared (NIR) spectra. In the present study, molecular absorption is taken into account through a Voigt line-shape model. The spectral range used for analyses of CO_2_ concentrations is 1568−1576 nm. As for the spectral data base, HITRAN2008 data base is used^3^. We use a spectrum analysis program that is basically the same as the previously reported one^2^. Since the molecular absorption lines in the NIR are pressure and temperature dependent, the spectral simulation requires a detailed representation of the vertical profiles of meteorological parameters.

From Goddard Earth Sciences Data and Information Services Center of NASA, we incorporate the Modern-Era Retrospective analysis for Research and Applications weather data products with observation time every minute at the geophysical location of the station with a km horizontal resolution. Here we briefly describe how the *X*CO_2_ mixing ratios are obtained. We assume homogeneous vertical profiles of mixing ratios from 0 to 48 km altitudes. For spectral simulation first, we divide the air layer into 28 sublayers. The sublayers were divided as follow: for every 1 km at altitude up to 16 km, 2 km at altitude 16−32 km, and 4 km at altitude 32−48 km. Next, we are applying the Beer-Lambert law to CO_2_ absorption in each layer for a certain CO_2_ molar mixing ratio. By summing up their absorbance with line-by-line calculation, we obtain the corresponding total absorbance for the CO_2_ molar mixing ratio. To obtain the column-averaged dry-air molar mixing ratios, the column densities of CO_2_ are divided by total column densities of dry air estimated from humidity weather data and reanalysis surface pressure^4^. The total column density of dry air is obtained by subtracting the mass of water vapor based on the reanalysis weather data of the relative humidity of the ground and the upper atmosphere. Air masses of the layers are calculated from the solar zenith angles (*SZA*) obtained from the GPS time and geography data of the station. There is a slight SZA dependency in the *X*CO_2_ data. For this dependency we test a correction formula similar to that implemented by Wunch *et al.*^5^. The correction influences only slightly our results for daily average values since the deviation are calculated to be less than 0.1%. With use of calibration curves thus obtained, the observed optical densities are converted to *X*CO_2_ as shown in Fig. S7-3.


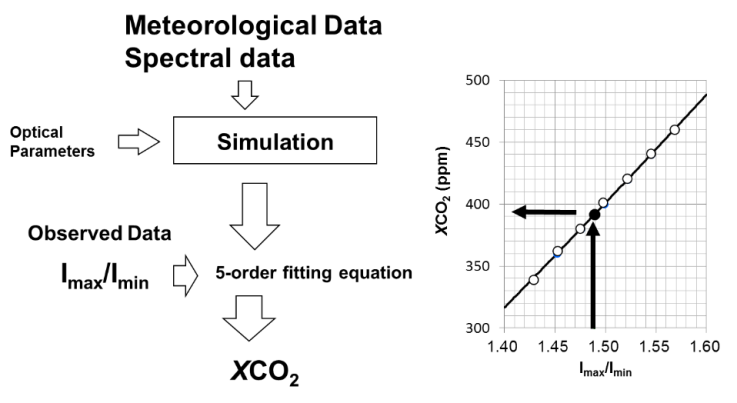


Figure S7-3. Example of calibration curves for conversion of observed optical density to *X*CO_2_(Power Point 2016)

***Comparison of instrument sensitivity*** The *X*CO_2_ data of FES-C were compared with a co-located TCCON FTS instrument between 1^st^ April−31^st^ May, 2014 at National Institute for Environmental Studies (NIES) at Tsukuba, Japan (36.05°N, 140.12°E). Since unstable weather or occasional thin-cloud coverage reduced reliability of the FES-C data, we eliminated data points of occasionally cloudy days mostly in April. Thus selected data points are 40. Day-average data for 40 days are plotted in Fig. S7-4. Referring to the FTS data, the FES-C scale factor is obtained:

*X*CO_2_(scaled) = *X*CO_2_(retrieved)/0.996

One standard deviation (1σ) of the scale factors is 0.0046.

Figure S7-4. Daily averaged data for solar time 10-14 h: (**○**) FTS at NIES, (●) scaled FES-C , Tsukuba, Japan from March to July, 2014. The vertical bars represent one sigma. (Power Point 2016)

***Column averaging Kernels*** Figure S7-5 shows column averaging Kernels of FES-C at Palangka Raya, Indonesia. Generally, a column averaging kernel represents a vertical profile of sensitivity of gas concentration changes at each atmospheric layer (height) to the column averaged gas concentration. If CO_2_ concentration is not vertically uniform, an XCO_2_ value retrieved depends on both profiles of CO_2_ concentration and averaging kernel. However, as the maximum variation of SZA during our measurements is 37.37° for the SZA change of 39.30° to 1.93° in Palangka Raya, the present variation ranges of sensitivity to XCO_2_ are expected to be less than 1% even if CO_2_ plumes flow only in the lower troposphere. Consequently, our measurements on XCO_2_ are not affected by the variation of SZA of measurements.


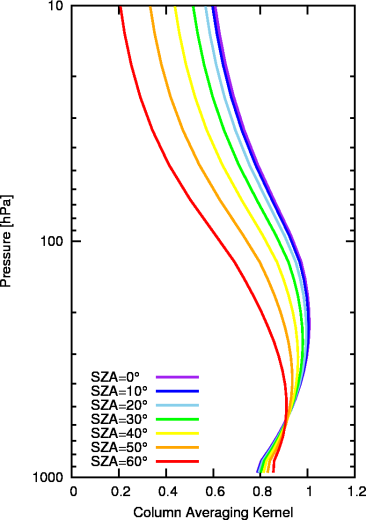


Figure S7-5. Column averaging Kernels of FES-C at Palangka Raya, Indonesia, on 28^th^ March, 2015

**Text S7 References**

1. Wilson, E. L, Georgieva, E. M. & Heaps, W. S. Development of a Fabry-Perot interferometer for ultra-precise measurements of column CO_2_. *Meas. Sci. Technol.* **18**, 1495–1502, doi: 10.1088/0957-0233/18/5/040 (2007).
2. Kobayashi, N. *et al*. Remotely operable compact instruments for measuring atmospheric CO_2_ and CH_4_ column densities at surface monitoring sites. *Atmos. Meas. Tech.* **3,** 1103–1112, doi: 10.5194/amt-3-1103-2010 (2010).
3. Rothman, L.S. *et al.* The HITRAN 2008 molecular spectroscopic database . *J. Quant. Spectrosc. Radiat. Trans.* **110**, 533–72, doi: 10.1016/j.jqsrt.2009.02.013 (2009).
4. Washenfelder, R. A. *et al*. Carbon dioxide column abundances at the Wisconsin Tall Tower site. *J. Geophys. Res. Atmos.* **111**, 1–11, doi: 10.1029/2006jD007154 (2006).
5. Wunch, D. *et al*. Calibration of the Total Carbon Column Observing Network using aircraft profile data. *Atmos. Meas. Tech*. **3**, 1351–1362, doi: 10.5194/amt-3-1351-2010 (2010).
